# A clinical series of packing the wound tract for arresting traumatic hemorrhage from injuries of the lung parenchyma as a feasible damage control technique

**DOI:** 10.1186/s13017-019-0273-y

**Published:** 2019-11-28

**Authors:** Alberto F. Garcia, Ramiro Manzano-Nunez, Juan Gabriel Bayona, Mauricio Millan, Juan C. Puyana

**Affiliations:** 1 Department of Surgery, Fundación Valle del Lili, Cali, Colombia; 2 Clinical Research Center, Fundación Valle del Lili, Cali, Colombia; 3 CISALVA Institute and Department of Surgery, Universidad del Valle, Cali, Colombia; 4 Department of Surgery, University of Pittsburgh, Pittsburgh, PA USA

**Keywords:** Wounds and injuries, Packing, Thoracic injuries, Damage control surgery

## Abstract

**Background:**

Tractotomy has become the standard of care for transfixing through-and-through lung injuries as it can be performed quickly with little blood loss and a low risk of complications. However, packing with laparotomy pads could be a feasible alternative to tractotomy on selected patients. We describe a series of four patients with lung trauma in which packing of the pulmonary wound tract was used as the primary and unique surgical strategy for arresting hemorrhage from injuries of the lung parenchyma.

**Methods:**

Packing of the traumatic tract is achieved by gently pulling a laparotomy pad with a Rochester clamp and adjusting it to the cavity to stop the bleeding. The pack is removed in a subsequent surgery by moistening and tractioning it softly to avoid additional damage. The operation is completed by manual compression of the wounded lobe. We present a case series of our experience with this approach.

**Results:**

From 2012 to 2016, we treated four patients with the described method. The mechanism was penetrating in all them. The clinical condition was of exsanguinations with multiple sources of hemorrhage. There were three patients with peripheral injuries to the lung and one with a central injury to the pulmonary parenchyma. Bleeding was stopped in all the cases. Three patients survived. A patient had recurrent pneumothorax which was resolved with a second chest tube.

**Conclusion:**

Packing of the traumatic tract allowed rapid and safe treatment of transfixing through-and-through pulmonary wounds in exsanguinating patients under damage control from several bleeding sources.

## Background

The tenets of damage control surgery were popularized by Rotondo et al. [1] in 1993. They established the feasibility of the damage control approach as an intervention to improve outcomes in severely injured patients. Since then, damage control surgery has become increasingly accepted as the standard of care, as it provides a survival benefit among injured patients with physiologic derangements.

The wide acceptance of damage control surgery for abdominal trauma allowed the translation and application of the concept to vascular [2] and chest trauma [3, 4]. Within the fields of thoracic trauma, the surgical treatment of pulmonary wounds has evolved toward more conservative procedures such as tractotomy and packing. Nowadays, most of the lesions are managed by tractotomy, which is associated with less morbidity and mortality [5–7]. This procedure minimizes local trauma and speed-up the procedure, which allows faster hemorrhage control [3, 4, 8].

Packing has been used to successfully control bleeding from the thoracic wall, from oozing surfaces or the surgical wound [9–11]. Despite the potential benefit of being a life-saving procedure, there remains a paucity of evidence on the feasibility of the use of thoracic packing as a damage control technique in the management of lung trauma. As of the writing of this paper, only two reports describe the use of pulmonary packing as a management strategy for injuries of the lung parenchyma [3, 12]. We describe a series of four patients with lung trauma in which packing of the pulmonary wound tract was used as the primary and unique surgical strategy for arresting hemorrhage from injuries of the lung parenchyma.

## Methods

### Setting and patients

The present report is a clinical case series of the use of packing of the pulmonary wound tract in critically injured patients. The cases described in these series were treated at la Fundacion Valle del Lili (FVL) University Hospital in Cali, Colombia from 2012 to 2016. FVL is equivalent to a US level I trauma center and admits more than 300 trauma patients with an ISS higher or equal to 15 per year [13].

We included all trauma patients who presented to our center and underwent surgery with packing of the pulmonary wound tract as the primary intervention for stopping the bleeding from injuries of the lung parenchyma. The cases described in these report were conducted as a matter of routine clinical care, and data was obtained from FVL medical charts.

### Indications for damage control surgery with packing of the pulmonary wound tract

During the study period, trauma patients who arrived to the emergency room were managed by the same group of trauma surgeons following institutional protocols. The decision to perform damage control surgery was based on early evidence of physiological exhaustion or the presence of multiple bleeding sources [3]. The method for arresting the hemorrhage was chosen according to the anatomy of the wound and the physiologic condition of the patient. In brief, isolated lobar injuries were managed with pulmonary tractotomy, pneumorrhaphy, and wedge resection.

In selected cases, packing of the pulmonary wound tract was the primary method of bleeding control; however, we acknowledge that this technique is not the standard of care in our center, and packing of the pulmonary wound tract was performed sporadically in patients physiologically exhausted and with more than one source of bleeding. Empiric observations on behalf of the treating trauma surgeon were that the lung tract packing technique was used as a desperate measure to transiently stop the pulmonary hemorrhage, while simultaneously, other lesions were treated.

The lung tract packing technique was used in transfixing central or peripheral penetrating wounds to the lung in patients who required surgical management following damage control principles. These are wounds which otherwise would have been treated with a tractotomy or a lung resection.

Damage control for thoracic lesions is best used for physiologically exhausted patients with multiple sources of bleeding, often outside of the thorax [3, 10–12, 14]. In these situations, the surgeon must choose the most straightforward and quickest procedure to speed-up the surgery and thus, stop the bleeding without inducing any additional tissue trauma.

### Packing of the pulmonary wound tract: technique description

Access to the thoracic cavities is most frequently gained by a fifth space anterolateral thoracotomy.

**Fig. 1:**
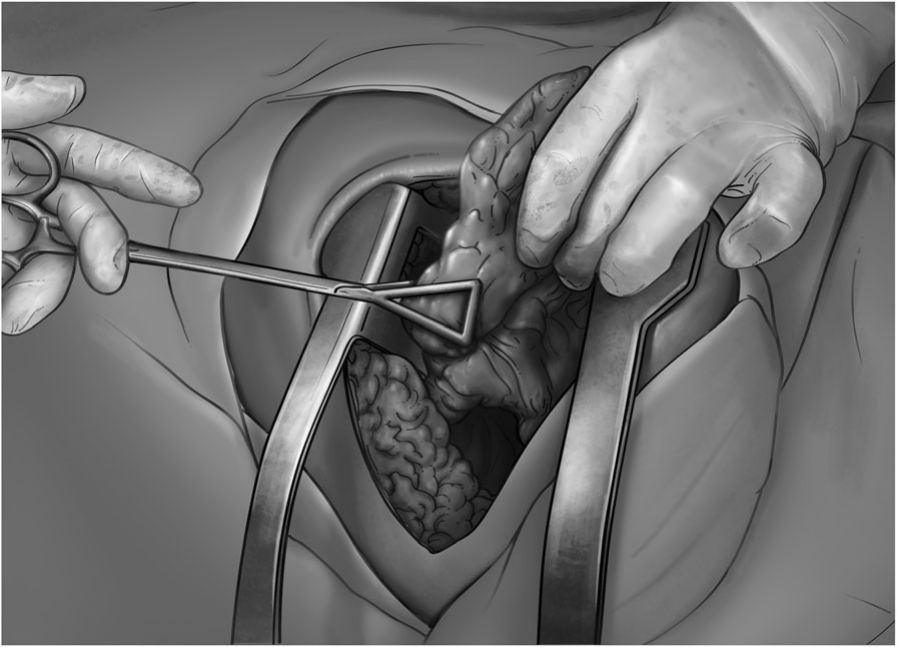
Temporary bleeding control by hand-collapsing the wounded lobe

Transient control of pulmonary bleeding depends on the location of the wounds. Bleeding arising from central or multi-lobar injuries is best managed by initially clamping the pulmonary hilum. Peripheral wounds are controlled by local compression, either through manual compression that collapses and compresses the entire lobe or by using Duval forceps (Fig. 1). At this point, the surgeon must decide whether to continue the surgery using traditional techniques, or whether to perform a damage control procedure. As previously mentioned, damage control principles should be followed if there is evidence of physiological exhaustion or the presence of multiple bleeding sources.

**Fig. 2:**
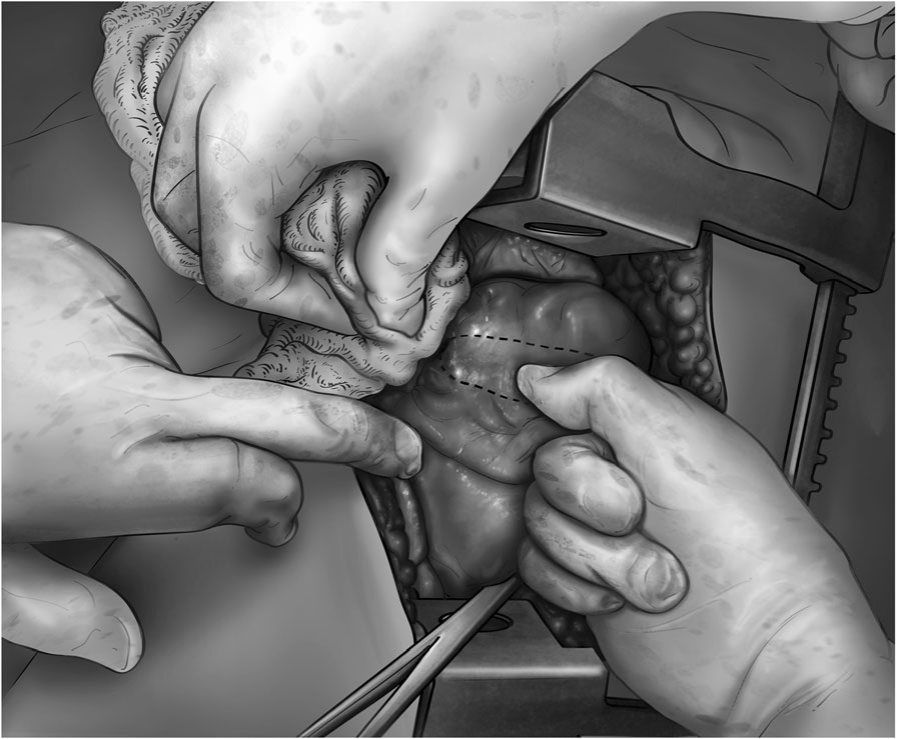
Finger exploration of the tract: the finger goes into the hole, gently exploring it, and preparing for the introduction of the clamp

**Fig. 3:**
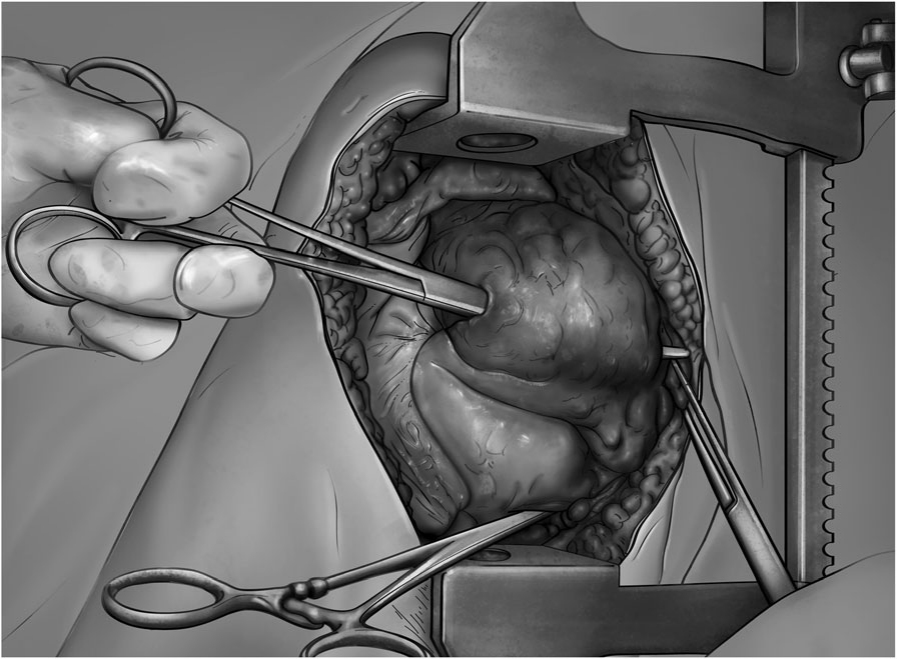
The Rochester clamp has been introduced through the tract

If damage control surgery with packing of the pulmonary wound tract is chosen, the surgeon introduces a finger into the tract, while compressing the tissue with the rest of the hand, to control bleeding (Fig. 2). After this, a Rochester forceps is guided into the tract in the direction opposite to the fingers, guided by the tip of the finger (Fig. 3).

**Fig. 4:**
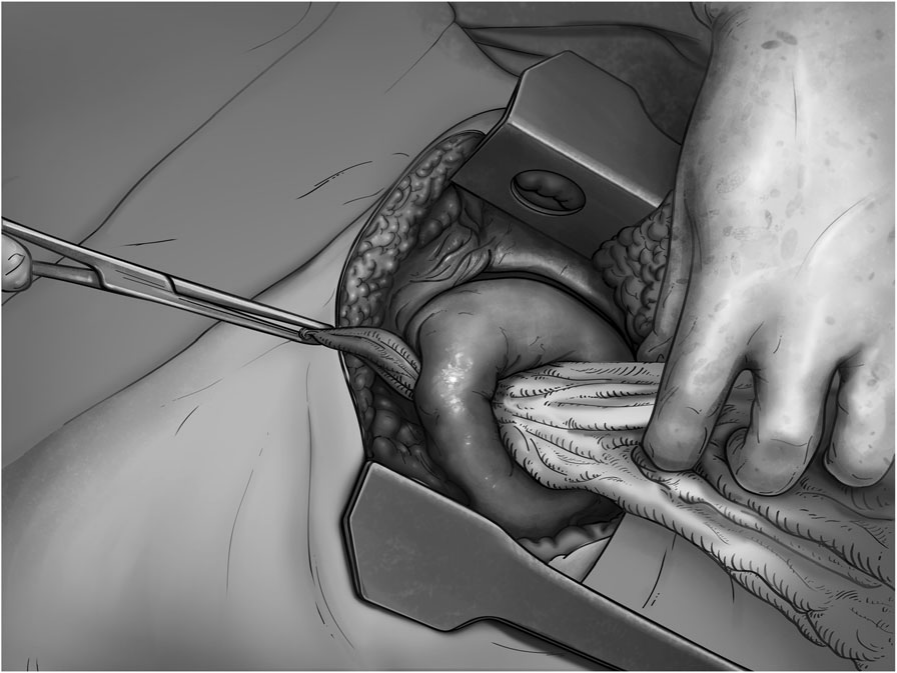
The pad is introduced into the tract. Frequent change in the direction of the traction helps to accommodate the pad into the wound

**Fig. 5:**
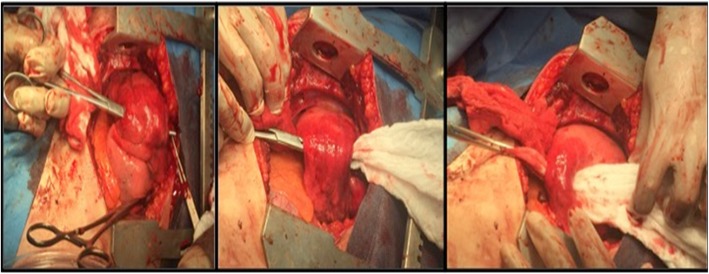
Packing of the wound tract in a patient with a transfixing lung injury

Once introduced, the clamp is used to pull a laparotomy pad through and into the tract. On the other hand, the surgeon exerts soft contra-traction to place the pad inside the wound (Figs. 4 and 5). The hemostatic effect is achieved by firm manual compression of the packed lobe. Redundant portions of the pad are folded around the lobe.

With hemorrhage control achieved and at the conclusion of surgery, a chest tube is left in the posterior recess, and the thoracotomy is closed by packing the muscular layers and the subcutaneous fat with two or three laparotomy pads and by suturing the skin above them with running monofilament stitches. This temporary closure technique for the thoracotomy wound takes 1 or 2 min and allows simultaneous control of the coagulopathic bleeding from the muscular layers.

After the surgery is completed, the patients are transferred to the ICU for close monitoring and resuscitation. Once physiologic exhaustion is controlled, the patients are taken back to the operating room 1 or 2 days later for unpacking and definitive chest wall closure. For the procedure, the thoracotomy is reopened, the thorax unpacked, and any bleeding is controlled. The pad in the tract is moistened with warm saline and carefully pulled back. The surgeon holds the lobe and gently pulls out the pad. The direction of traction is modified with the fingers, to minimize additional trauma.

After complete removal of the pad, and to achieve full hemostasis, the lobe is compressed for 2 or 3 min. Then, the surgeon must evaluate the presence of residual bleeding and any possible lack of air; to this end, the lung is allowed to expand. The persistence of significant bleeding or lack of air is indicative of the need for an additional procedure, such as tractotomy or resection. If neither of these is required, the thorax can be closed conventionally.

## Results

Packing of the pulmonary wound tract was used in four patients (Table 1). All of them were male; all arrived in hemorrhagic shock and required emergent surgery. All four patients had additional sources of bleeding, which required other surgical maneuvers. Packing of the tract, as previously described, was used as a temporary measure to permit the execution of more complex and critical interventions, or as an attempt to definitively control the bleeding in desperate situations.

**Table 1:** Characteristics of the patients managed with lung tract packing

Case	Age	Sex	Mec	ISS	Lung AIS	Thoracic lesions	Extra thoracic lesions	Reop	Outcome	Number of bleeding sources
1	20	M	GSW	25	5	ML, RIL	–	1	Live	2
2	19	M	SW	19	4	LSL, LIL	Subclavian artery	1	Death	3
3	36	M	GSW	25	4	LSL, LIL, BTW	Spinal cord injury	1	Live	3
4	21	M	GSW	75	3	RIL	Liver grade VI, colon grade II, SB grade IV	1	Live	3

*Mec*, trauma mechanism; *ISS*, injury severity score; *AIS*, abbreviated injury score; *Reop*, number of reoperations; *M*, male; *GSW*, gunshot wound; *SW*, stab wound; *ML*, middle lobe; *RIL*, right inferior lobe; *LSL*, left superior lobe; *LIL*, left inferior lobe; *BTW* bleeding thoracic wall; *SB*, small bowel

Patient 1 was found to have through-and-through wounds of the right medium and inferior lobes. The right pulmonary hilum was initially clamped, and a right medium lobe tractotomy was performed. At the moment of inferior lobe repair, the trauma surgeon found that the wound was deemed too central for a tractotomy. Therefore, packing of the wound tract was performed. Upon hilar clamp release, no recurrence of bleeding occurred, and thus the chest was left open and packed, and the patient was transferred post-op to the ICU.

Patient 2 suffered multiple stab wounds to the left side of zone I of the neck and the left hemithorax as well as the left thoracoabdominal region. The patient was found to have a left subclavian artery injury that was primarily repaired, and four stab wounds of the lung, two in the superior lobe and two in the inferior lobe. These lesions were initially treated with primary sutures, with apparent success. Six hours after index surgery, the patient had to be re-operated for persistent bleeding from the chest tube. He was found to have a 5-cm wound of the superior lobe with active bleeding. This injury was reopened, and the tract was filled with a laparotomy pad and subsequently closed, with adequate control of hemorrhage. Despite control of all surgical bleeding, the patient continued deteriorating and eventually died in the ICU.

Patient 3 was found to have through-and-through injuries to the superior and inferior left lobes and an injury to the intercostal vessels at the level of the posterior 9th intercostal space, where the bullet exited the thoracic cavity. A tractotomy succeeded in controlling the wound in the superior lobe. At this moment, the surgeon found persistent bleeding from the intercostal vessels which directed his efforts to achieve hemorrhage control from this source. Therefore, and as a temporary measure, the tract was packed with lap-pads. Once the control of intercostal vessels was achieved, the surgeon turned his attention to the inferior lobe injury. However, he found that packing successfully arrested hemorrhage and thus decided to end the procedure, left the chest open, packed, and send the patient to the ICU.

Patient 4 was admitted due to a right thoracoabdominal gunshot wound. Upon admission, FAST revealed blood in the right hemithorax and the abdomen. During the laparotomy, bleeding from the liver was controlled with peri-hepatic packing, colon wounds were sutured, and an ileum segment containing four perforations was resected and ligated with umbilical tape. A right anterolateral thoracotomy was performed due to continuing drainage of blood through the chest tube. He was found to have a through-and-through hole of the right inferior lobe and a diaphragmatic perforation from which dark blood was coming from the abdomen. The tract of the pulmonary wound was packed, the abdomen reopened, and a wound of the right suprahepatic vein was controlled with tight packing of the exit wound in segment IVa of the liver. Peri-hepatic packing was, therefore, used. Control of all surgical bleeding was achieved, and both the thorax and abdomen were left open, packed, and the patient was transferred to the ICU.

In all cases, packing of the pulmonary wound tract stopped the hemorrhage and the air leak. Patients 1, 3, and 4 survived and underwent unpacking after “triad of death” (i.e., acidosis, hypothermia, and coagulopathy) correction, which occurred after 2 days in the ICU in all cases. During the procedure, the pad was removed carefully, without causing further tissue damage. None of the survivors required additional surgical procedures such as tractotomy or resections to control hemorrhaging or air leaks during second-look operation. Moreover, the chest was closed during this operation, and no additional thoracic surgeries were required.

Only in one patient (case 4), a late air leak occurred, with a recurrent right pneumothorax that was treated with a second chest tube. Finally, upon hospital discharge, neither pneumonia nor other chest complications, and no additional adverse events were observed.

## Discussion

This case series report the safety and feasibility of the use of packing of the pulmonary wound tract as the primary and sole method to control bleeding from injuries of the lung parenchyma. We describe the technique of packing the pulmonary tract and present four patients in whom this procedure successfully stopped the bleeding and allowed the surgeon to direct his or her efforts to the management of the more complex existing lesions. Furthermore, the empiric observations on behalf of the treating trauma surgeons were that in every case, the decision to pack the tract of the pulmonary wound saved time and avoided additional blood loss and tissue damage.

Strategies to decrease morbidity and mortality in patients with severe lung trauma have evolved toward a damage control dogma. Nowadays, the damage control approach includes rapid control of bleeding and air leaks, delaying the treatment of non-life-threatening injuries during the initial operation, transferring patients to intensive care units for continued resuscitation, and a “*second-look*” surgery to complete the initial procedure, when the patient is in a better condition. In the last few decades, surgical treatment of pulmonary trauma has evolved toward less destructive methods. To date, there are only a few series that describe the use of thoracic damage control strategies. Thoracic packing has been reported [3, 10, 12, 15] but the use of packs to control lung parenchyma bleeding has only been mentioned in two publications [3, 12]. In these reports, packing with lap-pads was used as a complement to other hemostatic efforts such as ligation of the bleeding point, tractotomy, or resections. By contrast, in our series, packing was employed as the primary and unique maneuver to stop the bleeding from injuries of the lung parenchyma.

Packing with lap-pads is a mainstay of arresting non-compressible hemorrhage. It is a rapid and straightforward maneuver available to the surgeon who is dealing with a dying patient. Despite the usefulness of packing to control bleeding, some authors have suggested the deployment and inflation of a Foley catheter in the tract to achieve tamponade and hemostasis [16]. We prefer to use a lap-pad to fill the tract, instead of a catheter with a balloon such as those used to control the hemorrhage in traumatic liver tracts [17, 18]. First, it seems more natural to accommodate a pad to the conic cavity, rather than using a cylindrical balloon. Second, the low pressure required to collapse the pulmonary vessels is easily obtained with the gauzes, while filling the entire tract with the balloon may generate unnecessarily high pressures, which can disrupt the pulmonary tissue.

Through-and-through injuries to the lung may lead to some complications. First, the vasculature deep inside the tract will continue to bleed and form a hematoma which may finally form an abscess. Second, open bronchial injuries can produce post-operative air leaks. In our series, none of the patients developed a retained hemothorax or empyema. However, an air leak occurred in one case. Patient 4 had a recurrent pneumothorax that required a second chest tube. Leaks were reported after tractotomy in 36% of patients in Wall’s series [8], of which 20% was classified as prolonged. Velmahos et al. [5] reported an air leak in 9% of their patients, and Karmy Jones in 7% of patients treated with minor lung repairs [6].

## Limitations

Although our experience was successful, our observations in these patients should be interpreted cautiously. The information presented comes from a highly selected population, which may limit the translation of our results to other environments. Moreover, the small sample size of this case series (*n* = 4) under powers the probability of detecting the harms associated with the procedure when these harms exist, and thus, make it hard to draw meaningful conclusions with regard to the technique safety. Therefore, the favorable outcomes reported have a high probability of being due to chance.

Despite these limitations, we present a simple technical maneuver that is biologically plausible and allowed rapid bleeding control in patients with severe lung trauma. This technique could be useful in austere environments where resources are scare and the priority is to stop the bleeding.

## Conclusion

The lung tract packing technique should be considered an alternative option that is less destructive than other procedures, especially in desperate cases with multiple sources of hemorrhage when damage control is required. Although this method has been applied to only a limited number of patients, we found that packing of the pulmonary parenchyma successfully controlled the bleeding. Therefore, we consider this to be a promising approach.

## Data Availability

Data is property of the authors and can become available by contacting the corresponding author.
